# Real‐World Efficacy Profile of Compassionate Use of Asciminib in an Italian, Multi‐Resistant Chronic‐Phase Chronic Myeloid Leukemia (CML‐CP) Patient Population

**DOI:** 10.1002/hon.70101

**Published:** 2025-05-09

**Authors:** Massimo Breccia, Antonella Russo Rossi, Valentina Giai, Bruno Martino, Carmen Fava, Mario Annunziata, Elisabetta Abruzzese, Gianni Binotto, Claudia Baratè, Aurelio Pio Nardozza, Alessandra Misto, Paola Coco, Valeria Calafiore, Maria Cristina Carraro, Federica Cattina, Francesco Cavazzini, Maria Teresa Corsetti, Lara Crucitti, Monica Crugnola, Ambra Di Veroli, Paolo Ditonno, Anna Ermacora, Felicetto Ferrara, Angelo Genua, Antonella Gozzini, Stefana Impera, Alessandra Iurlo, Luciano Levato, Luigia Luciano, Maria Cristina Miggiano, Marco De Gobbi, Marco Santoro, Barbara Scappini, Anna Rita Scortechini, Andrea Patriarca, Serena Rosati, Sabina Russo, Rosaria Sancetta, Grazia Sanpaolo, Teresa Maria Santeramo, Silvia Sibilla, Federica Sorà, Paolo Sportoletti, Fabio Stagno, Elena Trabacchi, Fausto Castagnetti

**Affiliations:** ^1^ Division of Hematology Department of Translational and Precision Medicine Azienda Policlinico Umberto I Sapienza University Rome Italy; ^2^ Hematology and Transplantation Unit University of Bari Bari Italy; ^3^ Division of Haematology A.O.U. Città della Salute e della Scienza Turin Italy; ^4^ Division of Hematology Azienda Ospedaliera “Bianchi Melacrino Morelli” Reggio Calabria Italy; ^5^ Department of Clinical and Biological Sciences University of Turin Turin Italy; ^6^ Hematology Unit Cardarelli Hospital Napoli Italy; ^7^ Hematology Unit S. Eugenio Hospital ASL Roma2 Tor Vergata University Rome Italy; ^8^ Department of Medicine Unit of Hematology and Clinical Immunology University of Padova Padova Italy; ^9^ Department of Clinical and Experimental Medicine UO Hematology Azienda Ospedaliero Universitaria Pisana University of Pisa Pisa Italy; ^10^ Medical Department Novartis Novartis Farma SpA Milan Italy; ^11^ U.O. Oncoematologia A.O.R. Villa Sofia – Vincenzo Cervello Palermo Italy; ^12^ UOC Hematology and Transfusion Medicine Ospedale Luigi Sacco Milan Italy; ^13^ Oncology Unit Hospital Crema Crema Italy; ^14^ Hematology Unit University of Ferrara Ferrara Italy; ^15^ Hematology Division Azienda Ospedaliera Santi Antonio e Biagio e Cesare Arrigo Alessandria Italy; ^16^ Unità Operativa Ematologia Azienda Sanitaria Provinciale Trapani Presidio Ospedaliero Castelvetrano Trapani Italy; ^17^ Hematology Unit and BMT Center University Hospital of Parma Parma Italy; ^18^ Hematology Belcolle Hospital Viterbo Italy; ^19^ Haematology Unit National Cancer Center IRCCS Istituto Tumori “Giovanni Paolo II” Bari Italy; ^20^ Haematology Unit Azienda Ospedaliera S. Maria Angeli Pordenone Italy; ^21^ UOC Ematologia AORN “A. Cardarelli” Napoli Italy; ^22^ Hematology Unit Azienda Ospedaliera Santa Maria di Terni Terni Italy; ^23^ Department of Cellular Therapies and Transfusion Medicine AOU Careggi Florence Italy; ^24^ Division of Oncology and Hematology ARNAS Garibaldi‐Nesima Catania Italy; ^25^ Hematology Division Foundation IRCCS Ca' Granda Ospedale Maggiore Policlinico Milan Italy; ^26^ Department Hematology‐Oncology Azienda Ospedaliera Universitaria “Renato Dulbecco” Catanzaro Italy; ^27^ Hematology Unit Federico II University of Naples Naples Italy; ^28^ Hematology Department San Bortolo Hospital Vicenza Italy; ^29^ Department of Clinical and Biological Sciences Internal Medicine With Hematological Focus AOU San Luigi University of Turin Turin Italy; ^30^ UO Hematology University Hospital Paolo Giaccone Palermo Italy; ^31^ Hematology Unit Azienda Ospedaliero‐Universitaria Careggi Florence Italy; ^32^ Hematology Unit Azienda Ospedaliero Universitaria Ospedali Riuniti Ancona Ancona Italy; ^33^ Department of Translational Medicine Hematology Unit University of Eastern Piedmont and AOU Maggiore della Carità Novara Italy; ^34^ Ospedale Nuovo Santo Stefano Prato Italy; ^35^ Dipartimento di Patologia Umana dell'Adulto e dell'Età Evolutiva Division of Hematology Policlinico G. Martino University of Messina Messina Italy; ^36^ Hematology Unit ULSS 3 Dell'Angelo Hospital Venezia‐Mestre Italy; ^37^ Department of Hematology and Stem Cell Transplantation Unit IRCCS Casa Sollievo della Sofferenza Hospital San Giovanni Rotondo Italy; ^38^ Hematology Unit Dimiccoli Hospital Barletta Italy; ^39^ UO di Ematologia e trapianto Azienda Ospedaliera Cardinale G. Panico Tricase Italy; ^40^ Dipartimento di Scienze di laboratorio ed ematologiche Fondazione Policlinico Universitario A. Gemelli IRCCS Rome Italy; ^41^ Dipartimento di Scienze Radiologiche ed Ematologiche Sezione di Ematologia Università Cattolica del Sacro Cuore Rome Italy; ^42^ Centro Ricerche Emato‐Oncologiche Institute of Hematology Ospedale S. Maria della Misericordia University of Perugia Perugia Italy; ^43^ Hematology Unit AOU Policlinico “G. Martino” University of Messina Messina Italy; ^44^ Hematology Unit and BMT Center Ospedale G. Saliceto Piacenza Italy; ^45^ Institute of Hematology “Seràgnoli” IRCCS Azienda Ospedaliero‐Universitaria di Bologna Bologna Italy; ^46^ Department of Medical and Surgical Sciences University of Bologna Bologna Italy

**Keywords:** asciminib, chronic myeloid leukemia in chronic phase (CML‐CP), major molecular response (MMR), real‐world, TKI resistance/intolerance, tyrosine kinase inhibitor (TKI)

## Abstract

Chronic myeloid leukemia (CML) patients who have experienced failure and/or intolerance to multiple lines of treatment have limited therapeutic possibilities. Asciminib is a first‐in‐class tyrosine kinase inhibitor (TKI) that inhibits the ABL Myristoyl Pocket (STAMP or Specifically Targeting the ABL Myristoyl Pocket) within the *BCR::ABL1* oncoprotein. This retrospective Italian analysis reports the efficacy and safety outcomes of asciminib in treating 77 CML patients in chronic phase (CML‐CP) within a compassionate use setting. Patients were heavily pretreated with a median of 3 TKIs (55.8% had prior ponatinib exposure). Overall, 57.1% and 42.9% patients switched to asciminib because of resistance and intolerance, respectively. Asciminib maintained or improved molecular responses (MRs) in most patients: as best response, 41 patients (53%) achieved a MR3 or better, with 25 patients (32.5%) reaching deep molecular response (DMR). Greater percentages of intolerant patients achieved MR compared with resistant patients, although the probability of reaching at least a MR3 was not significant between the two groups (*p* = 0.116). Patients with the T315I mutation responded to asciminib, while ponatinib pre‐treated patients showed lower MR improvements compared to naïve patients and had a lower probability to reach a MR3 versus naïve patients (*p* = 0.0262). These results highlight asciminib remarkable tolerability and efficacy in real‐world CML‐CP patient population, including heavily pretreated patients, those intolerant and resistant to previous TKIs, and presenting several comorbidities.

**Trail Registration:**

The identification code for the MAP is CABL001AIT01M.

## 1 Introduction

Tyrosine kinase inhibitors (TKIs) have improved the prognosis of patients with chronic myeloid leukemia (CML), increasing overall survival (OS) globally. However, a substantial portion of patients interrupt the treatment due to resistance and/or intolerance [1, 2, 3, 4]. These conditions may arise when patients are treated with imatinib and after receiving second‐generation ATP‐binding TKIs [5, 6, 7, 8]. While the growing number of TKIs enables patients to receive multiple lines of therapies, the accumulation of new mutations with successive TKIs further increases the risk of treatment failure and reduces the sensitivity to the remaining TKIs [1, 3, 9]. Patients developing resistance and/or intolerance to multiple TKIs usually have worse clinical outcomes, showing an accelerated decrease in OS and response rates [1, 10].

Thus, resistance and intolerance to TKIs represent clinical challenges highlighting the urgent need for alternative therapeutic approaches for CML patients to prevent disease progression and treatment discontinuation.

Asciminib is the first‐in‐class TKI acting as an allosteric inhibitor on a different site (Specifically Target the ABL Myristoyl Pocket, or STAMP), involved in the autoregulation of ABL kinase activity [11, 12]. This mechanism of action is associated with retained activity against T315I and other *BCR::ABL1* mutations (except those located within the ABL myristoyl‐binding domain) and higher selectivity, allowing for reduced toxicity [11, 13, 14]. Asciminib has been developed to be potentially associated with other ATP‐competitive TKIs [15].

Asciminib efficacy and safety have been demonstrated in phase I (CABL001X2101) [11] and phase III (ASCEMBL) trials [12]. In the phase I trial, 69.6% of CML patients in chronic‐phase (CML‐CP) were still under treatment after a median exposure of approximately 4 years, and 61.6% reached or maintained major molecular response (MMR) (*BCR::ABL1* transcript ≤ 0.1%, also referred as MR3), confirming asciminib long‐term tolerability and efficacy [16]. The most recent follow‐up (median exposure duration: 5.9 years) showed that cumulative MMR rate continued to increase up to week 144; 23.6% and 18.9% of patients achieved MR4 and MR4.5, respectively, at week 96 [17]. CML‐CP patients harboring the T315I mutation received asciminib at 200 mg twice daily (BID); among them, 40.8% and 46.9% achieved MMR at 24 and 96 weeks, respectively [18]. Multiple trials are ongoing to further assess asciminib's efficacy and tolerability as monotherapy [19, 20] or in combination with imatinib [21, 22], dasatinib or nilotinib [22, 23].

ASCEMBL, a phase III multicenter, open‐label study, showed that in CML‐CP patients previously treated with ≥ 2 TKIs, asciminib provided a higher 24‐week MMR rate (25.5%) versus bosutinib (13.2%, *p* = 0.029) [12]. This favorable trend persisted for up to 156 weeks, with an MMR rate of 33.8% (versus 10.5% with bosutinib) and a good safety/tolerability profile [24]. Asciminib was approved in Europe in August 2022 for patients with Philadelphia chromosome‐positive (Ph+) CML‐CP who had previously received at least two TKIs [25].

Real‐life studies complement results from trials. An Italian analysis described a yearly increment of 97.6% in the number of patients treated with TKIs as ≥ 3rd lines [26], and the management of patients in later lines was associated with an increase in healthcare costs, primarily due to hospitalization [27]. These data emphasize the burden of the disease within the Italian clinical practice and reiterate the need for innovative therapies to improve CML approach in heavily treated subgroups.

We present real‐world evidence on the impact of asciminib in a cohort of 77 Italian TKI‐resistant and/or intolerant CML‐CP patients included in a Managed Access Program (MAP) approved by Novartis (CABL001AIT01M). This report aims to expand the current knowledge of asciminib effectiveness and safety for the management of CML‐CP patients treated with multiple lines of TKIs.

## 2 Materials and Methods

### 2.1 Patient Population

This analysis included 77 CML‐CP Ph+ patients receiving asciminib through a Novartis MAP, between April 2019 and October 2022, in 41 Italian institutions. These patients had experienced treatment failure to ≥ 2 TKIs due to resistance (MRs not reached at selected time points as per European Leukemia Network [ELN] 2020 recommendations [28]) and/or intolerance (unacceptable adverse events leading to treatment interruption). Patients were administered asciminib orally at a dosage of 40 mg BID, except those carrying the T315I mutation, who received a dosage of 200 mg BID as currently recommended (based on the asciminib phase I results [18]). The asciminib MAP was approved by ethical committees in each of the involved centers; written informed consent was obtained from all patients before asciminib initiation, following the local regulations and the Declaration of Helsinki.

Patients eligible for the treatment were adults > 18 years with a diagnosis of Ph+ CML who met one of the two following criteria: (i) CML‐CP in absence of the T315I mutation, and relapsed, refractory, or intolerant to at least 2 prior TKIs; (ii) have either CML‐CP with the T315I mutation, or accelerated/blastic phase CML resistant, intolerant or with contraindication to all available treatments.

Patients were excluded if they presented persistent platelet levels ≤ 50 × 10^9^/L, active or uncontrolled cardiovascular conditions, infections, uncontrolled pancreatitis or liver disease, or any other uncontrolled medical conditions. Patients under therapy with medications belonging to the CYP3A inducers or inhibitors category were also excluded.

### 2.2 Clinical Outcomes

Baseline patients' characteristics were obtained from the administrative database of all the centers involved in the MAP.

MR was collected at baseline and every 3 months during the follow‐up and reported according to the International Scale (IS) as *BCR::ABL1*% on a log scale, as indicated by the ELN 2020 recommendations (*BCR::ABL1* ≤ 10% = MR1; *BCR::ABL1* ≤ 1% = MR2; *BCR::ABL1* ≤ 0.1% = MR3/MMR; *BCR::ABL1* ≤ 0.01% = MR4; *BCR::ABL1* ≤ 0.0032% = MR4.5; *BCR::ABL1* ≤ 0.001% = MR5). All MR ≤ 0.01% (MR4, MR4.5 and MR5) were considered as deep MRs (DMR). *BCR::ABL1* analysis was conducted in EUTOS‐accredited laboratories. MR was monitored and analyzed according to ELN 2020 recommendations [28].

*BCR::ABL1* mutational analysis was evaluated as it is internationally recommended in case of resistance (absence of optimal response); mutational status was not assessed during or after asciminib treatment.

Event‐free survival (EFS) was defined as the time from first asciminib dose to treatment discontinuation for resistance/intolerance (ELN criteria) [28].

### 2.3 Statistical Analysis

Categorical variables are presented as absolute numbers and relative frequencies, and continuous variables as median with relative range (min, max). MR distribution is described at baseline, at 3 months, according to the best response, and at last follow‐up considering all treated patients, and patients with the T315I mutation only. Patients were analyzed according to (1) reason for discontinuation of the last TKI before asciminib (i.e., resistance and intolerance), (2) ponatinib condition (in all treated patients and patients with or without T315I mutation, separately). The proportion of patients with improved MR during asciminib treatment is also shown. The proportions of ponatinib‐naïve and pre‐treated patients, including the subgroup of those with T315I mutation, who reached at least MR3 from asciminib start, are compared using logistic regression model and providing the estimate of odds ratios (OR) and relative 95% confidence intervals (CI). Kaplan‐Meier curve is provided for the Event‐Free survival. All statistical analyses were done with SAS software 9.4 (SAS Institute, Cary, NC, USA).

## 3 Results

### 3.1 Patients' Demographic and Baseline Characteristics

Out of the 77 patients included in the analysis, 39 (50.6%) were males. The median age of the whole cohort was 63 years (range 20–85). More than half of the patients (51.7%) reported ≥ 3 comorbidities. Before starting asciminib, patients were heavily pretreated with a median of 3 TKIs (42.9%, 27.3% and 29.9% of patients received 2, 3 and ≥ 4 TKI lines, respectively), and the median time from diagnosis to asciminib initiation was 6 years (range 1–34). The switch to asciminib occurred for resistance in 44 patients (57.1%) and intolerance in 33 patients (42.9%). Median asciminib exposure time was 8.5 months (range 3–38). A total of 43 patients (55.8%) had prior exposure to ponatinib; of these, 38 (49.4%) had ponatinib as the last TKI before asciminib, with 19 patients switching due to resistance and 18 due to intolerance (data from one patient were missing).

At asciminib initiation, 24 patients (31.2%) harbored mutations in the ABL kinase domain; of them, 11 (45.8%) had the T315I mutation (3 had an isolated T315I mutation and 8 had T315I in association with other mutations) while 13 (54.2%) had only other mutations. All patients carrying the T315I mutation had prior treatment with ponatinib. These patients had a median value of mean daily dose of asciminib of 400 mg (range 68.9–400).

A summary of baseline patients' characteristics is reported in Table 1.

**TABLE 1:** Demographic and clinical characteristics at baseline. Data are expressed as *n* (%) or median (range).

Baseline characteristics (*n* = 77)	
Age, median (range)	63 (20–85)
Males, *n* (%)	39 (50.6)
Sokal risk at diagnosis, *n* (%)	
Low	14 (18.2)
Intermediate	18 (23.4)
High	27 (35.1)
Unknown	18 (23.4)
*BCR::ABL1* mutation, *n* (%)	
Wild type	53 (68.8)
Mutated	24 (31.2)
‐ Only T315I^a^	3 (12.5)
‐ Only other(s)^a^	13 (54.2)
‐ Both T315I and other mutation(s)^a^	8 (33.3)
Comorbidities, *n* (%)	
None	19 (24.7)
1	16 (27.6)
2	12 (20.7)
≥ 3	30 (51.7)
Prior use of ponatinib, *n* (%)	43 (55.8)
Years from diagnosis to asc initiation, median (range)	6.0 (1–34)
Asc exposure (months), median (range)	8.5 (3–38)
Last TKI before asc, *n* (%)	
Ponatinib	38 (49.4)
Dasatinib	12 (15.6)
Nilotinib	11 (14.3)
Bosutinib	10 (13.0)
Imatinib	6 (7.8)
Reason for last TKI discontinuation, *n* (%)	
Resistance	44 (57.1)
Intolerance	33 (42.9)
Reason for discontinuation, ponatinib as last TKI, *n* (%)	
Resistance	19 (24.7)
Intolerance	18 (23.4)
Missing	1 (1.3)
Number of TKI treatment lines before asciminib, overall population, median (range)	3 (2–7)
Patients receiving 2 lines of TKI, *n* (%)	33 (42.9)
Patients receiving 3 lines of TKI, *n* (%)	21 (27.3)
Patients receiving ≥ 4 lines of TKI, *n* (%)	23 (29.9)
Prior TKIs lines in ponatinib‐naïve patients	2 (2–5)
Prior TKIs lines in ponatinib pre‐treated patients	3 (2–7)
Best response with previous treatment lines, *n* (%)	
≤ MR1	16 (20.8)
MR2	23 (29.9)
MR3	14 (18.2)
DMR	23 (29.9)
Unknown	1 (1.3)
Median of mean asc daily dose (mg), patients without T315I mutation (range)	80.0 (23.2–80.0)
Median of mean asc daily dose (mg), patients with T315I (range)	400.0 (68.9–400)
Patients with dose modification, *n* (%)	13 (16.9)

^a^ Percentages are computed on mutated patients.

### 3.2 Efficacy, MRs Versus Baseline

**FIGURE 1:**
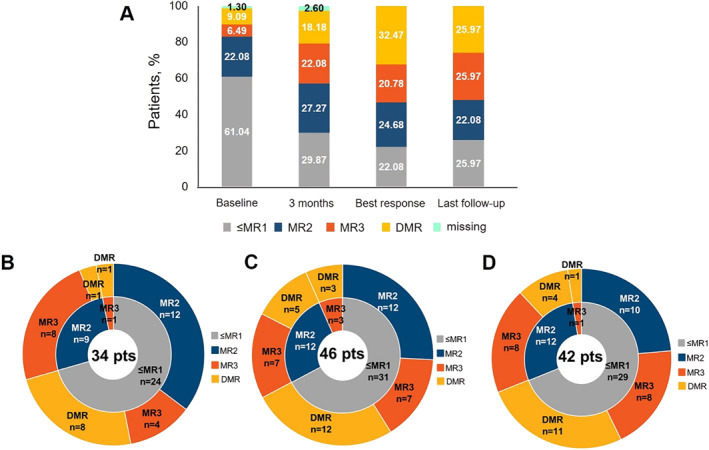
(A) Percentage of patients achieving ≤ MR1, MR2, MR3, DMR during the treatment with asciminib. Radial charts showing the change in MR from baseline among patients who achieved an improved response at 3 months (B), as best response (C) and at last follow‐up (D).

MR distribution is summarized at 3 months, as the best response (i.e., best MR observed) and at the last follow‐up (Figure 1A).

Patients with MR ≤ MR1 decreased at all time points versus baseline. The percentage of patients displaying either MR3 or DMR was greater than at baseline at all time points considered; as best response, 41 patients (53%) achieved MR3 or better, with 25 patients (32.5%) reaching a DMR.

Specifically, patients whose MR improved from baseline were 34 (44.2%) at 3 months, 46 (59.7%) as best response and 42 (54.5%) at last follow‐up (Figure 1B–D). The MR change from baseline was analyzed among patients with improved response at 3 months, best response, and last follow‐up (Figure 1B–D). Interestingly, 31 patients had ≤ MR1 at baseline and then, as best response, 12 reached MR2 (38.7%), 7 reached MR3 (22.6%) and 12 obtained DMR (38.7%). Of 12 patients having MR2, 7 achieved an MR3 and 5 a DMR; indeed, 3 patients started as MR3 and all achieved a DMR (Figure 1C).

**FIGURE 2:**
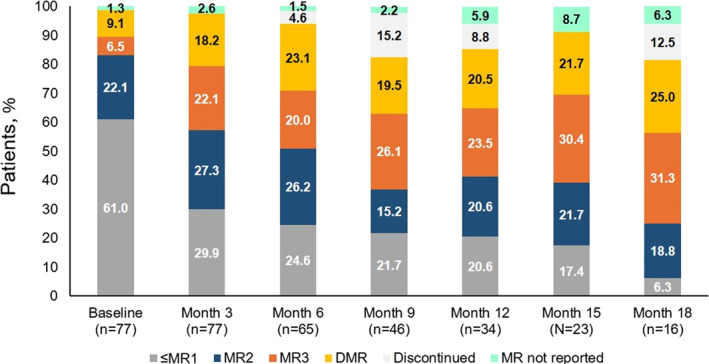
MR distribution including discontinued patients at each timepoint. Data were recorded up to 18 months of asciminib treatment. “MR not reported” refers to patients with missing MR at the timepoint of interest and with asciminib exposure longer than timepoint of interest. Each discontinued patient is shown at the first timepoint in which they discontinued the treatment and they are excluded from treated patients at all the subsequent timepoints.

Figure 2 represents the MR distribution at each timepoint also considering the proportion of patients who interrupted the treatment.

### 3.3 Efficacy, MRs Versus Baseline in Patients With Last TKI Discontinuation Due To Resistance or Intolerance

**FIGURE 3:**
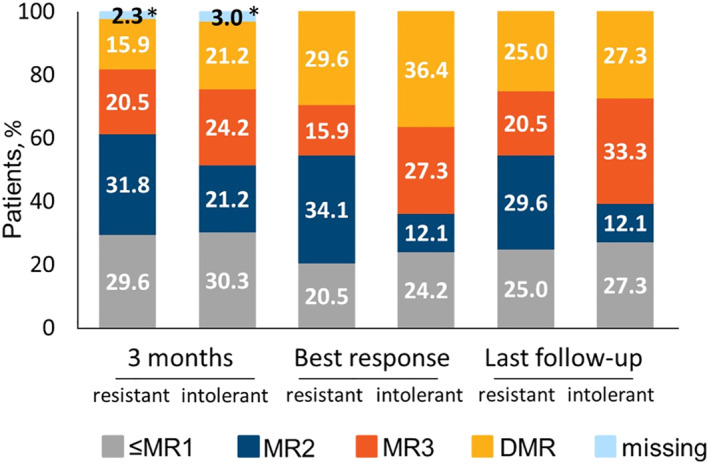
Percentage of resistant and intolerant patients achieving ≤ MR1, MR2, MR3, DMR during the treatment with asciminib. Data were recorded at 3 months, as best response and at last follow‐up. *One missing result was due to a MR value not available at baseline.

As shown in Figure 3, slightly greater percentages of intolerant patients achieved MR3 and DMR compared with TKI‐resistant patients at all time points. As best response, ≥ MR3 was achieved by 63.7% of intolerant patients (27.3% in MR3 and 36.4% in DMR) versus 45.5% resistant (15.9% in MR3 and 29.6% in DMR), resulting in an OR of 2.10 that showed a trend to reach at least MR3 response in intolerant versus resistant patients, although not statistically significant (95% CI: 0.83–5.29, *p* = 0.116).

### 3.4 Efficacy, MR Versus Baseline in Patients With T315I Mutation and According to Previous Ponatinib Use

Asciminib response was examined in patients harboring the *BCR::ABL1* T315I mutation (*n* = 11, 14.3%). At baseline, these patients showed either ≤ MR1 (*n* = 9, 81.8%) or MR2 (*n* = 2, 18.2%) (Supporting Information S1). Overall, 5 (45.5%) patients showed an improved MR at 3 months, 6 (54.6%) as best response, and 5 (45.5%) at last follow‐up. Most reached DMR (60% at 3 months, 66.7% as best response, and 80% at the last follow‐up).

Most patients were previously treated with ponatinib (*n* = 43, 55.8%); 38 patients (88.4%) received ponatinib as the last TKI before asciminib. To understand whether asciminib effectiveness was affected by previous ponatinib use, we compared MR in ponatinib pre‐treated and naïve patients. Overall, ponatinib‐naïve patients improved baseline MR to a greater extent compared with pre‐treated patients: 52.9% versus 37.2% at 3 months, 76.5% versus 46.5% as best response, 70.6% versus 41.9% at last follow‐up (Supporting Information S1). Accordingly, ponatinib pre‐treated patients had a lower probability of reaching ≥ MR3 versus naïve patients (OR: 0.34, CI 95%: 0.13–0.88, *p* = 0.0262).

Among the 34 ponatinib pre‐treated patients without MR3 (*BCR::ABL1* > 0.1%) at baseline, 12 (35.3%) reached MR3 in a median time of 2.9 months (range: 0.3–12.0), whereas among the 30 ponatinib‐naïve patients without MR3 at baseline, 19 (63.3%) reached an MR3 in a median time of 3.9 months (range: 0.5–7.8) (Supporting Information S1). Among the 19 patients with ponatinib as the last TKI who switched because of resistance, 2 achieved a MR3 and 7 a DMR as best response starting from MR1/MR2 at baseline (Supporting Information S1). Among the 18 ponatinib‐intolerant patients, 4 achieved a MR3, and 4 a DMR as best response (Supporting Information S1).

Similar results were observed in patients without the T315I mutation (Supporting Information S1). Within this subgroup, patients pre‐treated with ponatinib had a lower probability of achieving ≥ MR3 than ponatinib‐naïve patients, but the difference was not significant: OR = 0.25 (95% CI: 0.06–1.02, *p* = 0.0527).

### 3.5 Asciminib Persistence and Discontinuation

Median exposure to asciminib treatment was 8.5 months (range 3–38). A total of 60 patients (77.9%) continued the treatment, while 17 (22.1%) interrupted asciminib. The reasons for discontinuation were allogenic transplant (*n* = 7, 9.1%), progression of the disease (*n* = 5, 6.4%), resistance (*n* = 2, 2.6%), intolerance (*n* = 2, 2.6%), or death (*n* = 1, 1.3%). Among patients with disease progression, two harbored the T315I mutation along with additional mutations at baseline (G250E, E255K, E255V, T315L, T315M, E459K in one case, F359I, M244V, E255K in the other), with progression occurring at 3 months and after 3 years of asciminib treatment, respectively. One patient with a baseline E499E mutation developed a new F539 V mutation and progressed at 9 months, while two patients without detectable mutations progressed after 6 months.

Of the two patients discontinuing because of resistance, one without detectable mutations stopped treatment after 6 months in MR2, while the other, with a baseline Y253H mutation, discontinued after 6 months with a response below MR1 and no new mutations.

**FIGURE 4:**
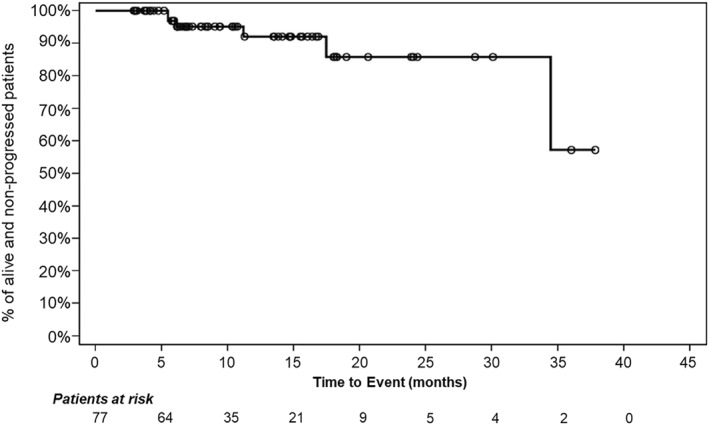
Kaplan‐Meier curve showing the event‐free survival of patients treated with asciminib. Dots represent censors. Patients at risk at each timepoint are those who are not censored and have yet to experience the event of interest.

Figure 4 reports the Kaplan‐Meier curve showing event‐free survival (EFS).

### 3.6 Adverse Events

A total of 45 adverse events (AEs) were reported, 15.6% hematological and 84.4% non‐hematological. The most common hematological AE was thrombocytopenia (11.2%), probably related to asciminib and was generally manageable without dose modification. The most frequent non‐hematological AEs were gastrointestinal disorders (8.9%) and lipase/amylase increase (4.4%), probably related to asciminib (Table 2).

**TABLE 2:** All‐grade AEs (hematological and non‐hematological) occurring during asciminib treatment.

Total adverse events (*n*)	45
Hematological adverse events^a^, *n* (%)	7 (15.6)
Preferred term (MedDRA), *n* (%)	
Thrombocytopenia	5 (11.2)
Platelet increased	2 (4.4)
Non‐hematological adverse events^a^, *n* (%)	38 (84.4)

SOC (MedDRA)	Preferred term (MedDRA)	*n* (%)
Gastrointestinal disorders (*n* = 4, 8.9%)	Dyspepsia	1 (2.2)
	Diarrhea	1 (2.2)
	Nausea	1 (2.2)
	Vomiting	1 (2.2)
Investigation (*n* = 3, 6.7%)	Hyperbilirubinemia	1 (2.2)
	Lipase increase	1 (2.2)
	Amylase increase	1 (2.2)
Musculoskeletal and connective tissue disorders (*n* = 6, 13.3%)	Myalgia	2 (4.4)
	Arthralgia	1 (2.2)
	Bone pain	1 (2.2)
	Osteonecrosis	1 (2.2)
	Undifferentiated connective tissue disease	1 (2.2)
Infections and infestations (*n* = 4, 8.9%)	COVID‐19	2 (4.4)
	Rhinitis	1 (2.2)
	Urinary tract infection	1 (2.2)
Neoplasms benign, malignant and unspecified (*n* = 7, 15.6%)	Malignant neoplasm progression	4 (8.9)
	Basal cell carcinoma	1 (2.2)
	Brenner tumor	1 (2.2)
	Other myeloproliferative neoplasm	1 (2.2)
Other (*n* = 14, 31.1%)	Hemorrhage	3 (6.7)
	Respiratory failure	3 (6.7)
	Angina pectoris	1 (2.2)
	Peripheral artery stenosis	1 (2.2)
	Chest pain	1 (2.2)
	Pyrexia	1 (2.2)
	Hyperhidrosis	1 (2.2)
	Paresthesia	1 (2.2)
	Renal failure	1 (2.2)
	Decrease appetite	1 (2.2)

^a^ Cases reported here refer only to spontaneous reporting, therefore the number/rate of AEs could be underestimated.

## 4 Discussion

This analysis evaluates asciminib effectiveness and safety in CML‐CP patients within the compassionate use setting, and includes a substantial proportion of patients treated with asciminib as ≥ 3rd TKI line. Asciminib response has been examined at three time points; MRs have been assessed in the overall population and in subgroups of patients based on intolerance/resistance to previous TKIs, presence of the T315I mutation and previous ponatinib use. In previous studies, asciminib effects were evaluated in CML populations who were predominantly in CP, but with a minor proportion of patients in either accelerated phase (up to 9% [29]) or blastic phase (up to 3% [30]).

Our population featured a high level of comorbidities and was extensively treated with prior TKIs. Despite these unfavorable characteristics, most patients improved their baseline MR as best response and at last follow‐up. Remarkably, of patients with MR1 at baseline, 39% obtained DMR as best response. Overall, these results are in line with previous studies; Kockerols et al. showed that asciminib improved the response in up to 66% of patients who did not have MMR at baseline [29], and up to 32% achieved MR4 or better in the British study by Innes and colleagues [31].

Patients who exhibited intolerance or resistance to prior TKIs responded positively to asciminib. As expected, higher percentages of intolerant patients achieved MMR and DMR compared to resistant patients, although the OR to achieve MR3 as best response was not significant. Previous real‐life studies [30, 32] showed greater effectiveness of asciminib among intolerant versus resistant patients, reporting even a more pronounced discrepancy [32]. These data underscore asciminib's therapeutic advantage in TKI intolerant patients and the clinical challenge of treating resistant patients. Nevertheless, in our report, 30% and 16% of resistant patients showed MR3 or DMR, respectively, as best response.

Asciminib inhibits the proliferation of cell lines carrying various *BCR::ABL1* mutations, including T315I [13, 14], known for conferring resistance to TKIs [33, 34]. Phase I trial demonstrated that patients harboring the T315I mutation maintained or achieved MMR when treated with asciminib at 200 mg BID [18]. Therefore, we employed the same dose in patients harboring the T315 mutation. Our data show that 54.6% of these patients improved MR as best response, highlighting asciminib's therapeutic value in this subgroup. While previous real‐life studies have shown positive effects of asciminib in this context, the number of patients with the T315I mutation was generally low with heterogeneous results [32, 35, 36]. Kockerols et al. evaluated the largest cohort of CML‐CP with the T315I mutation in real life (*n* = 12); of these, most were already in MR4 at asciminib initiation and maintained MR4 throughout the treatment, while none of the 4 remaining patients reached ≥ MR3 [29].

Ponatinib is a third‐generation ATP‐competitive TKI whose function is preserved in patients with T315I mutation [34, 37]. However, other *BCR::ABL1* mutations are known to confer resistance to the treatment [38, 39], and ponatinib use has been linked with a high risk of cardiovascular events [40]. Although to date no head‐to‐head studies are available, an indirect analysis [41] suggests asciminib offers at least comparable MMR improvement to ponatinib. In our analysis, most patients had prior exposure to ponatinib. When comparing ponatinib pre‐treated to naïve patients, pre‐treated exhibited a lower response to asciminib. The same difference was observed when analyzing only patients without the T315I mutation. Asciminib superiority in ponatinib‐naïve versus pre‐treated patients was reported in the phase I trial [18] and real‐life studies [29, 35, 36]. Of note, Luna et al. found asciminib to be most effective in intolerant, ponatinib‐naïve patients, but showed a similar response in resistant patients regardless of prior ponatinib use [36]. Similarly, Kockerols et al. observed a worse response in patients with primary ponatinib failure, whereas patients who discontinued ponatinib for intolerance with MR ≥ 2 maintained MR during asciminib treatment [29].

Considering the short observation period, our data show asciminib discontinuation rate of 22%, which is favorable compared to previous studies (27% in the phase I study [11] and 25%–28.5% in real‐world studies [32, 36]). Most patients interrupted asciminib for disease progression; only 2.6% developed either resistance or intolerance. Asciminib was overall well tolerated, with most AEs occurring in less than 10% of cases. Thrombocytopenia, commonly found in asciminib‐treated patients [32, 36], developed in 11% of patients.

Our findings expand current knowledge on asciminib effectiveness and safety in a large real‐life CML‐CP patient population by assessing the patients' responses at distinct time points, both in the overall population and within subgroups. Compared to other studies [32, 36], our analysis includes a substantial number of ponatinib pre‐treated patients and patients with the T315I mutation. The main limitation is its retrospective nature; moreover, a higher number of patients would have been necessary to conduct additional analyses. Future studies should be designed prospectively and with longer follow‐ups.

## 5 Conclusions

Asciminib is a compelling therapeutic option for CML‐CP patients, even outside of clinical trials, demonstrating a remarkable effectiveness and tolerability in patients treated with ≥ 2 TKIs and with multiple comorbidities. Patients with the T315I mutation also benefited from asciminib treatment.

## Author Contributions

All authors critically reviewed and revised the manuscript and approved the final version for submission. All authors took final responsibility for the integrity of the data, the accuracy of the data analysis, and the decision to submit the manuscript for publication.

## Ethics Statement

The asciminib MAP was approved by ethical committees in each of the involved centers. The treating physicians obtained written informed consent from all participants or their representatives prior to the start of treatment, in accordance with the local laws and regulations and in line with the ethical principles outlined in the Declaration of Helsinki. Confirmation of informed consent was communicated to Novartis through the Managed Access System prior to the treatment initiation.

## Consent

Written informed consent was obtained from all patients prior to asciminib treatment initiation.

## Conflicts of Interest

A.P.N., A.M., P.C. are Novartis employees.

### Peer Review

The peer review history for this article is available at https://www.webofscience.com/api/gateway/wos/peer-review/10.1002/hon.70101.

## Permission to Reproduce Material From Other Sources

The authors have nothing to report.

## Supporting information

Supporting Information S1

## Data Availability

The data that support the findings of this analysis are available from the corresponding author on reasonable request.
