# Supplementary material for: Real‐World Efficacy Profile of Compassionate Use of Asciminib in an Italian, Multi‐Resistant Chronic‐Phase Chronic Myeloid Leukemia (CML‐CP) Patient Population
Source: Hematol Oncol. 2025 May 9;43(3):e70101. doi: 10.1002/hon.70101 (PMC12064212; doi:10.1002/hon.70101)
Supplement: Supplementary file 1 — Supporting Information S1 [file HON-43-e70101-s001.docx]

**Real-world efficacy profile of compassionate use of asciminib in an Italian, multi-resistant chronic-phase chronic myeloid leukemia (CML-CP) patient population**

Massimo Breccia, Antonella Russo Rossi, Valentina Giai, Bruno Martino, Carmen Fava, Mario Annunziata, Elisabetta Abruzzese, Gianni Binotto, Claudia Baratè, Aurelio Pio Nardozza, Alessandra Misto, Paola Coco, Valeria Calafiore, Maria Cristina Carraro, Federica Cattina, Francesco Cavazzini, Maria Teresa Corsetti, Lara Crucitti, Monica Crugnola, Ambra Di Veroli, Paolo Ditonno, Anna Ermacora, Felicetto Ferrara, Angelo Genua, Antonella Gozzini, Stefana Impera, Alessandra Iurlo, Luciano Levato, Luigia Luciano, Maria Cristina Miggiano, Marco De Gobbi, Marco Santoro, Barbara Scappini, Anna Rita Scortechini, Andrea Patriarca, Serena Rosati, Sabina Russo, Rosaria Sancetta, Grazia Sanpaolo, Teresa Maria Santeramo, Silvia Sibilla, Federica Sorà, Paolo Sportoletti, Fabio Stagno, Elena Trabacchi, Fausto Castagnetti

**Table 1S. MR evaluation in patients with T315I mutation during the treatment with asciminib.**

| MR in patients with T315I mutation, n (%) | | | | |
| --- | --- | --- | --- | --- |
|  | **≤MR1** | **MR2** | **MR3** | **DMR** |
| Baseline (n=11) | 9 (81.8) | 2 (18.2) | – | – |
| MR at 3 months (n=11*) | 5 (45.5) | 1 (9.1) | 1 (9.1) | 3 (27.3) |
| Best response (n=11) | 5 (45.5) | 1 (9.1) | 1 (9.1) | 4 (36.4) |
| Last follow-up (n=11) | 6 (54.6) | – | 1 (9.1) | 4 (36.4) |

*One missing result was due to a MR value not available at 3 months

**Table 2S. Patients who reached MR3 by ponatinib condition.**

| Patients who reached MR3 by ponatinib condition, n (%) | | |
| --- | --- | --- |
|  | Naïve (n=34) | Pre-treated (n=43) |
| Patients with MR<MR3 at baseline | 30 (88.2) | 34 (79.1) |
| Patients who reached MR3^a^ | 19 (63.3) | 12 (35.3) |
| Median time to reach MR3^b^, months (range) | 3.9 (0.5–7.8) | 2.9 (0.3–12.0) |

^a^ Percentages were computed on patients who reported MR<MR3 at baseline, i.e., MR='≤MR1' or MR='MR2’

^b^ Only for patients who reached MR3.

**Figure 1S. Percentage of patients with improved response *vs* baseline, by ponatinib condition (naïve and pre-treated).** A: overall treated population (n=77, naïve n=34, pre-treated=43). B: patients without T315I mutation (n=66, naïve n=34, pre-treated n=32).

* Missing results were due to a MR value not available at baseline or at month 3; ^a^ n=34; ^b^ n=43; ^c^ n=32


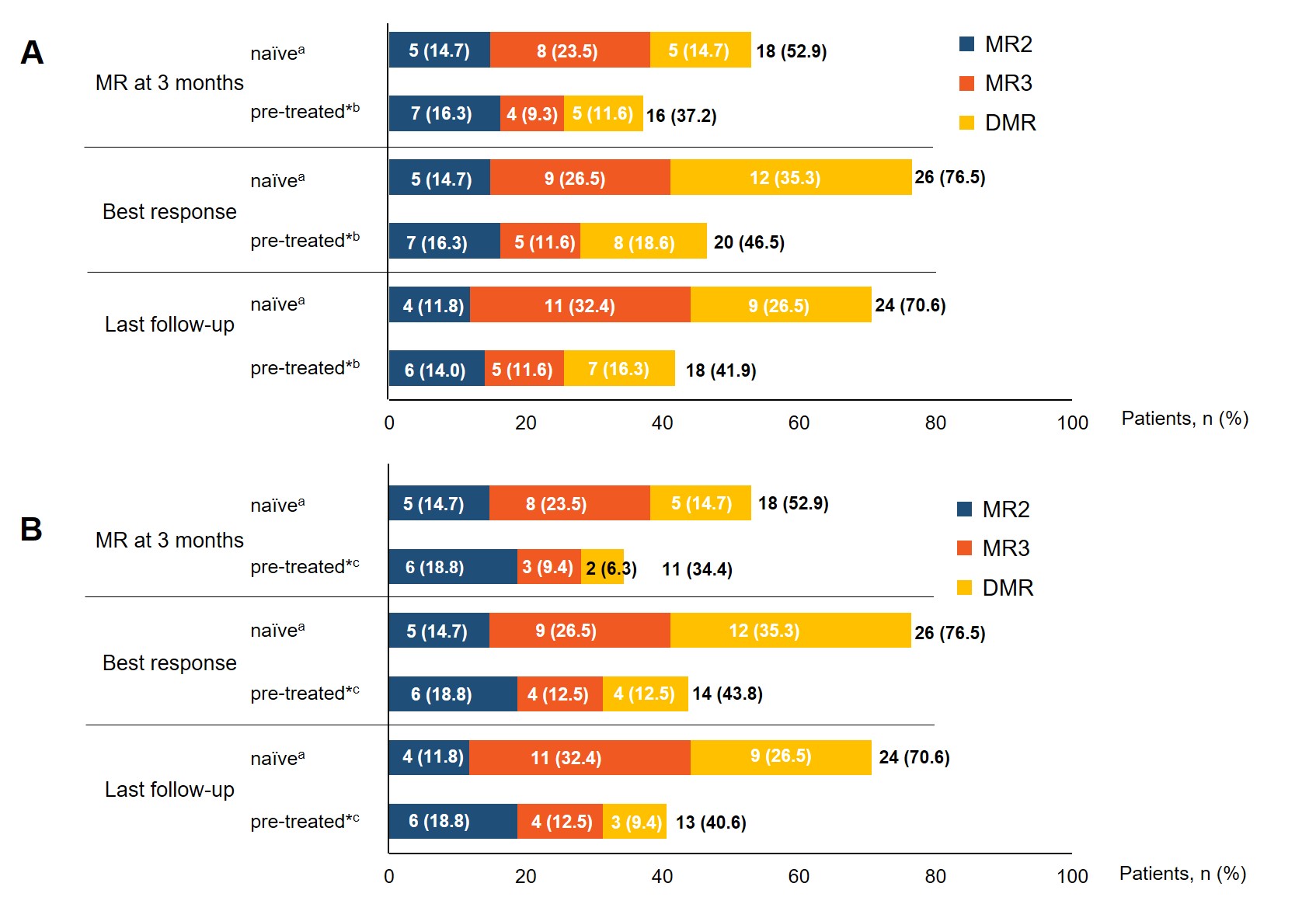


**Figure 2S.** Radial charts showing the change in baseline MR among patients with ponatinib resistance (A) or intolerance (B) as best response.

**
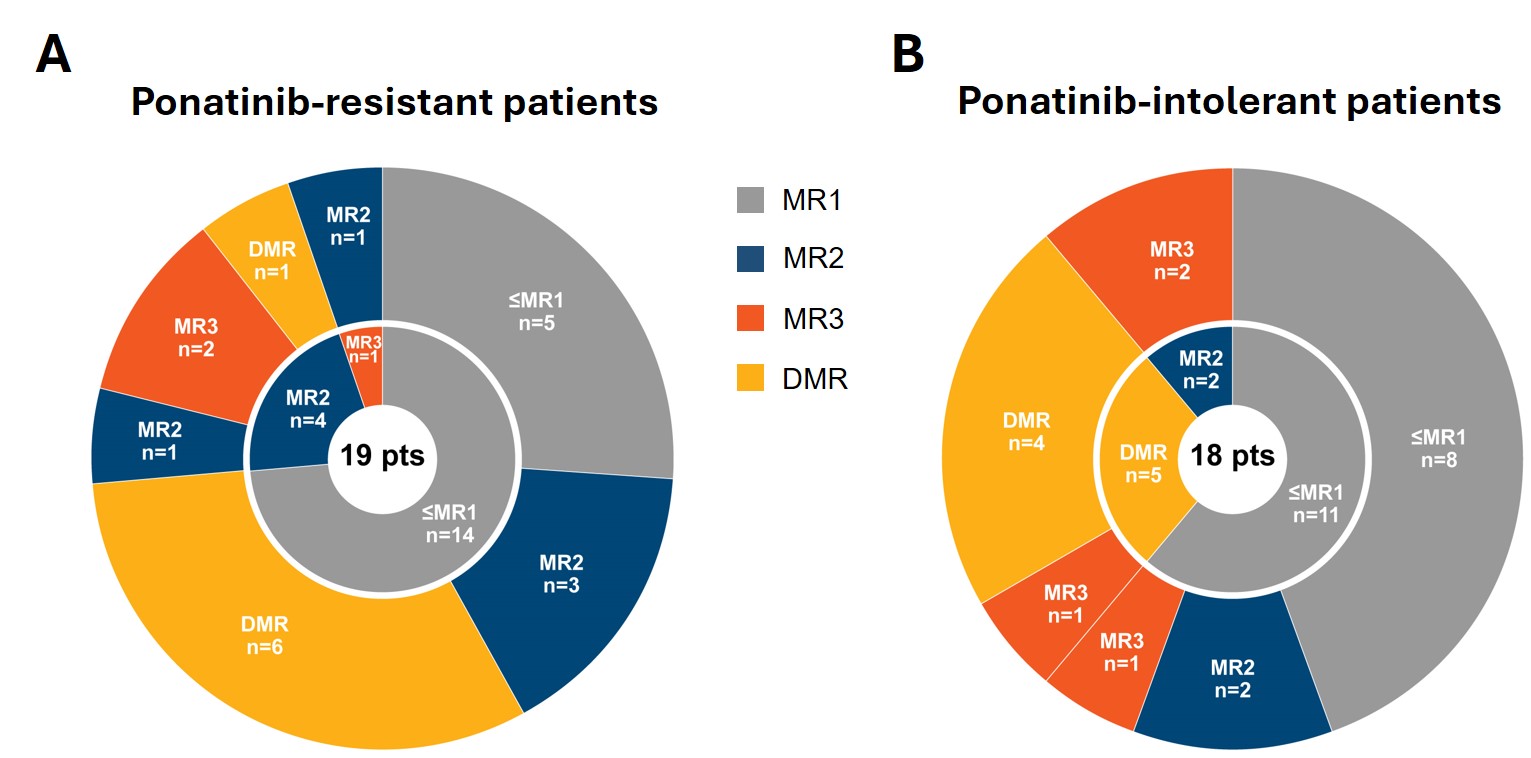
**
